# Option Grids to facilitate shared decision making for patients with Osteoarthritis of the knee: protocol for a single site, efficacy trial

**DOI:** 10.1186/1472-6963-14-160

**Published:** 2014-04-07

**Authors:** Katy Marrin, Fiona Wood, Jill Firth, Katharine Kinsey, Adrian Edwards, Kate E Brain, Robert G Newcombe, Alan Nye, Timothy Pickles, Kamila Hawthorne, Glyn Elwyn

**Affiliations:** 1 Cochrane Institute of Primary Care and Public Health, School of Medicine, Cardiff University, Neuadd Meirionnydd, Heath Park, CF14 4YS Cardiff, UK; 2 Pennine MSK Partnership Ltd, Integrated Care Centre, New Radcliffe Street, Oldham OL1 1NL, UK; 3 South East Wales Trials Unit, School of Medicine, Cardiff University, Neuadd Meirionnydd, Heath Park, Cardiff CF14 4YS, UK; 4 The Dartmouth Center for Health Care Delivery Science, Dewey Field Road, Hanover, NH 03755, USA

**Keywords:** Shared decision making, Decision aids, Osteoarthritis of the knee, Arthritis, Health literacy, Patient communication

## Abstract

**Background:**

Despite policy interest, an ethical imperative, and evidence of the benefits of patient decision support tools, the adoption of shared decision making (SDM) in day-to-day clinical practice remains slow and is inhibited by barriers that include culture and attitudes; resources and time pressures. Patient decision support tools often require high levels of health and computer literacy. Option Grids are one-page evidence-based summaries of the available condition-specific treatment options, listing patients’ frequently asked questions. They are designed to be sufficiently brief and accessible enough to support a better dialogue between patients and clinicians during routine consultations. This paper describes a study to assess whether an Option Grid for osteoarthritis of the knee (OA of the knee) facilitates SDM, and explores the use of Option Grids by patients disadvantaged by language or poor health literacy.

**Methods/Design:**

This will be a stepped wedge exploratory trial involving 72 patients with OA of the knee referred from primary medical care to a specialist musculoskeletal service in Oldham. Six physiotherapists will sequentially join the trial and consult with six patients using usual care procedures. After a period of brief training in using the Option Grid, the same six physiotherapists will consult with six further patients using an Option Grid in the consultation. The primary outcome will be efficacy of the Option Grid in facilitating SDM as measured by observational scores using the OPTION scale. Comparisons will be made between patients who have received the Option Grid and those who received usual care. A Decision Quality Measure (DQM) will assess quality of decision making. The health literacy of patients will be measured using the REALM-R instrument. Consultations will be observed and audio-recorded. Interviews will be conducted with the physiotherapists, patients and any interpreters present to explore their views of using the Option Grid.

**Discussion:**

Option Grids offer a potential solution to the barriers to implementing traditional decision aids into routine clinical practice. The study will assess whether Option Grids can facilitate SDM in day-to-day clinical practice and explore their use with patients disadvantaged by language or poor health literacy.

**Trial registration:**

Current Controlled Trials ISRCTN94871417

## Background

There is significant policy interest and an established ethical imperative for involving patients in decisions about their healthcare [1,2]. In some medical conditions there is clear evidence that a treatment is highly effective, and the need for comparing alternative approaches is negligible. However, in many other conditions the balance of benefit to harm of a range of treatment options may be much less obvious, and the choice of treatment will be dependent on the preferences of the individual patient, in consultation with their practitioner. In such situations, shared decision making (SDM), an approach where clinicians and patients make decisions together using the best available evidence, is appropriate [3].

Patient decision support interventions, often called decision aids, have been developed to support SDM between practitioners and patients. These tools differ from traditional information resources in several ways: they make options explicit rather than imply a preferred choice; they use the best available evidence to be clear about benefits and harms; and many are interactive, making use of media such as the web and DVDs. Evidence suggests that patients who participate in SDM with the help of these tools are more knowledgeable about treatment options, make decisions that are more consistent with their own attitudes toward benefits and harms, and have more informed discussions with their practitioners [3,4].

Despite wide policy interest, the promotion of SDM has not led to sustained use in routine practice [5]. A significant problem has been the narrow interpretation of SDM, as if the provision of tools alone could somehow automatically lead to better communication between clinicians and patients about treatment options [6]. The reality is that these approaches do not seem to work well [7,8]. Professionals resist their use in practice, and are ambivalent about the idea of sharing decisions with patients [9,10]. Practical barriers include the time required to implement SDM supported by decision aids in routine practice, consequently causing disruption to clinic workflows. Typically, the tools have been quite extensive and can take time for a patient to read or view them, as well as requiring a degree of technical and health literacy [11]. They are costly to develop and producers often have commercial pay walls, which limit their accessibility [3].

Little is known about using patient decision support tools in populations that are ethnically diverse or socio-economically disadvantaged. Very few tools have been specifically designed for low-literacy populations [12,13]. Decision tools have predominantly been developed in English and often contain information that may be difficult for people with lower levels of literacy, and for those who do not have English as their first language [14].

Work to address the implementation challenges faced by decision aids has resulted in the development of brief decision making tools designed to be used in the clinical encounter called Option Grids [5]. Drawing on decision theories that refer to heuristics or “rules of thumb” [15], Option Grids are one-page evidence-based summaries of the available condition-specific treatment or screening options, presented in a tabular format, listing patients’ essential trade-offs or frequently asked questions [5,16,17]. The underpinning theory and rationale for Option Grids, their development process and guidance for their use in the clinical encounter, is reported elsewhere [5,16]. Option Grids are deliberately brief, free of clinical jargon, and can be read by an individual with a reading age of 10 to 12 years. Formatted to one page and hosted on an open access website (http://www.optiongrid.org), they can be downloaded and printed with minimal cost [5].

The Option Grid website (http://www.optiongrid.org) currently hosts 28 Option Grids and considerable interest has been shown in them by the medical community, with thousands of Option Grid downloads since the website’s inception in 2012. Evidence from an implementation study suggests that clinicians find these short tools helpful as they remove the burden of having to talk extensively about options, and they notice that this structured information exchange takes either less, or no additional, time [13]. Patient comprehension is facilitated by a layout that displays the alternative options side-by-side for easy comparison. Patients are enabled to raise relevant personal issues, picking the frequently asked questions as their starting point [18].

The interest from the clinical community in these tools indicates their wish to explore the feasibility of Option Grids for supporting SDM, but evidence from controlled studies of their effects in day-to-day clinical situations is currently lacking. We therefore developed a controlled trial to test the efficacy of an Option Grid in facilitating SDM. A concurrent qualitative evaluation will explore patients’, physiotherapists’ and interpreters’ views on the acceptability of using Option Grids in clinical encounters.

### Objectives

The primary objective is to assess the impact of the Option Grid (for OA of the knee) on SDM in routine encounters, in comparison to usual care where the Option Grid was not provided. Secondary objectives are to: assess the impact of Option Grids on patient decision making preferences in a clinical setting; examine whether these tools lead to good outcomes for physiotherapists and patients; evaluate the feasibility of using Option Grids in routine clinical practice; assess whether these tools can also be used with groups disadvantaged by language and literacy barriers and the extent of their reach; and evaluate whether there is willingness among practitioners to embed these tools into clinical pathways.

## Methods

### Study design

**Figure 1:**
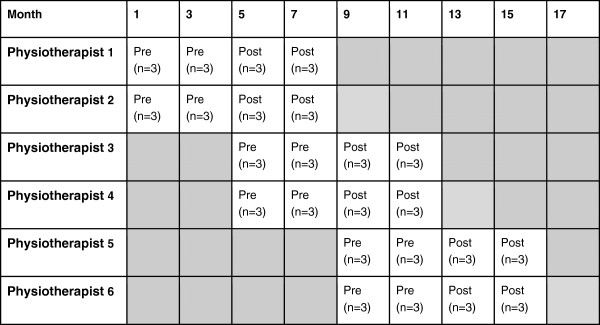
Physiotherapist allocation to the option grid.

This study is a stepped wedge design where the intervention, an Option Grid for OA of the knee, is allocated to six physiotherapists in a time controlled sequence (Figure 1) [19]. In a stepped wedge design, an intervention is rolled-out sequentially to the trial physiotherapists over a number of time periods. It is useful in situations where the intervention cannot be delivered concurrently to all units. A before and after evaluation will be undertaken, comparing outcomes for patients participating in the intervention time period with those in the control time period.

### Intervention

The intervention is the use of an Option Grid for OA of the knee by a trained physiotherapist, where the tool acts as a basis for the comparison of treatment options, and further discussion. The standardised development process for the Option Grids is documented elsewhere [19]. In the current study, the OA knee Option Grid was developed by a team of clinicians and academics with an interest in shared decision making, musculoskeletal conditions and pain management. User testing, to assess the language and formatting of the Option Grid, was undertaken via interviews and a focus group with staff and lay members of the charity Arthritis Care. This testing led to some changes in language and ranking of patients’ frequently asked questions.

**Figure 2:**
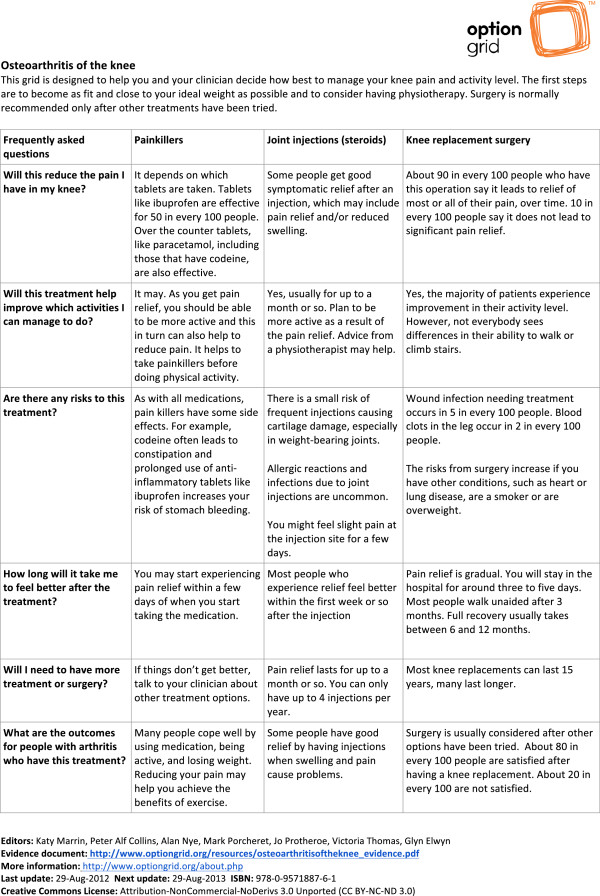
Option grid for osteoarthritis of the knee.

The aim of the Option Grid is to facilitate shared decision making (SDM) in routine consultations by prompting discussion about the patient’s key concerns [5]. The Option Grid presents three treatment options for OA of the knee in table format: painkillers; steroid injections into the joint space; and knee replacement surgery. OA knee patients’ frequently asked questions are listed on the side of the table, allowing users to compare answers across the three treatment options (Figure 2). Guidance on how to use Option Grids in routine consultation has been outlined elsewhere [5] and can be found on the Option Grid website (http://www.optiongrid.org/). Participating physiotherapists will receive brief (30 minutes) training on how to use the Option Grid within a routine consultation after having consulted with six patients using usual care procedures (control).

### Outcomes

The primary outcome is the efficacy of an Option Grid in facilitating SDM as measured by observer scores using the OPTION scale [20]. Secondary analysis will explore: the quality of decision making as measured by the Decision Quality Measure (DQM); treatment decision at three months; the extent to which the intervention has reached disadvantaged groups as measured by the literacy of participants utilising the REALM-R instrument [21] and socio-demographic variables; and participants’ pain severity using a Visual Analogue Score [22]. A qualitative investigation will explore the acceptability of the Option Grids to patients and physiotherapists, and how these tools are used in tripartite consultations where interpreters are present.

### Setting

This is a single site trial located in Pennine Musculoskeletal Partnership Ltd (PMSK Ltd) in Oldham, UK. PMSK have been contracted by Oldham Clinical Commissioning Group (CCG) to provide integrated services to the local population in rheumatology, orthopaedics and chronic musculoskeletal pain. The majority of patients are referred to the service by General Practitioners (GPs), where they can access a range of multidisciplinary specialist health services. Referrals have also been accepted from standard physiotherapy and PMSK nurses. Patients with knee problems usually attend a first consultation with an extended scope physiotherapist, who will undertake a diagnostic assessment and decide with the patient the most appropriate course of management for the condition, which may involve treatment such as joint injections, further assessment or referral to an orthopaedic surgeon, voluntary and independent sector providers, as required.

Oldham’s population is is ethnically diverse (22.5% non-white, compared to England as a whole 14.3%), has low rates of employment (58.3%, England 62.1%), and has a relatively high proportion of its population with no educational qualifications (29.6%, England 22.5%) [23]. We therefore considered this site to be suitable for assessing whether an Option Grid would be acceptable and could be used by patients who are disadvantaged by language and literacy.

### Sample size

10,000 new patient referrals a year are received by PMSK Ltd and it is estimated that 12-18 patients per month will present with OA of the knee which requires consideration of treatment options. Based on previous studies, the anticipated mean and standard deviation (SD) for the OPTION score are 16.9 and 7.7. Assuming that the effect of the intervention is to increase the mean by 50% and that the SD remains unaltered (the distribution simply shifts upwards) then the mean will increase to 25.35. With 36 patients (6 physiotherapists consulting 6 patients each) pre-intervention and 36 patient post-intervention, the expected value of *t* is 4.66 Adjusting for the clustering effect at the level of the physiotherapist (intra-cluster correlation = 0.22) results in a variance inflation factor (design effect) of 2.1, therefore the expected value of *t* changes to 3.22. This corresponds to a power, using this sample of 72 patients, of roughly 90% to detect a difference of this size. A 40% consent rate is estimated, requiring a population of 180 eligible patients to be contacted in order to obtain a sample of 72 patients. A conservative data collection phase of 18 months via a stepped wedge design is planned.

### Randomisation

Physiotherapists will be randomised to a starting time point, with physiotherapists paired to start trial procedures simultaneously, i.e. physiotherapists 1 and 2 will be allocated to start first (and thus receive the intervention first) and physiotherapists 5 and 6 will be allocated to start last. See Figure 1. Without excluding any patients, each physiotherapist will consult with six patients before they are provided with the intervention. After receiving training on how to deliver the intervention, the physiotherapists will consult with a further six patients.

### Eligibility and recruitment

Patients over the age of 18 years with OA of the knee who are referred to Pennine MSK are eligible for the trial. A research nurse, based in PMSK Ltd, will identify and contact eligible patients from GP referrals. Patients who agree to participate will be sent an information pack (comprising of an invitation letter, information sheet and consent form) with their appointment letter. Patients will be asked to provide informed written consent when they arrive for their appointment. Physiotherapists in the service will be briefed about the trial aims and objectives. Those who specialize in lower limb problems and indicate a willingness to participate will receive an information pack and written consent will be consented prior to data collection.

### Data collection

To obtain the observer OPTION Score using the Observer Option Scale, each consultation will be audio-recorded and assessed by two raters using the 12-item measure [20]. A mean of the two raters will be calculated. Immediately post consultation, patients will complete: a DQM, an adaptation of other decision quality scales (13 item scale of knowledge, preferences and intention) [24]; Visual Analogue Score (1 item scale of pain severity) [22]; and the REALM-R reading exercise (8 items) [21]. They will then participate in a semi-structured interview. Treatment decision at three months will be collected from medical records. Socio-demographic information (age, gender, postcode and highest educational attainment) will be collected from medical records and patient interviews. Physiotherapists will be interviewed twice: once before entering the trial, and once after they have completed their participation in the trial. Interpreters will be interviewed up to two weeks after each consultation in which they participated. Clinical encounters will be observed by the research nurse and a structured observation record will be made of each consultation.

### Analysis

Statistical analysis will be undertaken using IBM SPSS Statistics 20 and MLwiN 2.28. The primary outcome of interest is the efficacy of the Option Grids in terms of facilitating SDM, to be measured using the Observer OPTION Scale [20]. Pre and post-intervention Observer OPTION scores will be assessed using multilevel modelling, where the patient will be the first level and the physiotherapist will be second level. Secondary analysis of the DQM, using multilevel modelling, will be used to compare whether the intervention improves knowledge and improves the ability to make a choice. The treatment decision will be assessed to compare post-intervention to pre-intervention sample. Data on age, gender, educational level, employment status and language and the REALM-R score will provide information on the socio-demographic profile of study participants as well as the extent to which they are considered to be at risk of poor health literacy. The pain severity score will provide information about the trial population and allow the identification of associations between trial outcomes and pain severity of its participants as a potential effect modifier.

Observation notes, patient, interpreter and physiotherapist interview data will be transcribed and analysed using critical discourse analysis, supported by qualitative data analysis software NVIVO version 10, to explore views on the consultation, perceptions of involvement in decision making and the use of the Option Grid. Using a coding framework, one member of the research team will code all interview data, and another will dual code a 30% sample of interviews. A sample of audio-recorded consultations purposefully sampled on the basis of a range of Option Scores and REALM-R scores will be selected for transcription and will be studied in more detail using discourse analysis. Discourse analysis is particularly suited to the analysis of dialogue in a social interaction (physiotherapist-patient consultation). It allows researchers to interpret the meanings that people ascribe to the words they use, to expose identities and the inconsistencies and inequalities within social relationships [25].

### Ethical approval

The study protocol was approved by the South East Wales Research Ethics Committee (11/WA/0356). Written participant information about trial objectives and procedures will be given to eligible patients. Standard forms are used to document informed consent.

## Discussion

This trial will be the first to determine the efficacy of an Option Grid on facilitating shared decision making in day-to-day clinical encounters. If the intervention is found to facilitate shared decision making it will make a significant contribution to knowledge about how to promote SDM and overcome the implementation barriers experienced to date [7].

## Abbreviations

DQM: Decision quality measure; OA of the knee: Osteoarthritis of the knee; PMSK: Pennine musculoskeletal partnership Ltd; SDM: Shared decision making.

## Competing interests

The authors declare that they have no competing interests.

## Authors’ contributions

The study was conceptualised and designed by GE, who was awarded the BUPA Foundation grant to undertake the study. Up to January 2014, KM was the Trial Manager, helped design the study, undertook data collection and wrote the first draft of the paper. GE and FW are the Co-Chief Investigators, provided leadership for the project. AE, RN and JF contributed to the study design. KH is a former member of the Trial’s management group providing advice on black and minority ethnic (BME) issues; RN and TP are the trial statisticians. GE, AN, KM and contributed to the development of the intervention. KK undertook recruitment and data collection. All authors sit on the Trial’s management group and contributed to drafting the manuscript and approved the final manuscript.

## Authors’ information

KM is formerly Research Associate at the Cochrane Institute of Primary Care and Public Health, Cardiff University (currently Senior Consultant, Miller Research Ltd). KB, FW AE and RN are Senior Lecturers and Professors respectively at the Cochrane Institute of Primary Care and Public Health, Cardiff University. GE is a Professor and Senior Scientist at the Dartmouth Center for Health Care Delivery Science. KH is a Clinical Professor Associate Dean of Medical Education at Cardiff University; JF is Consultant Nurse in Rheumatology/Clinical Governance Lead at PMSK and Visiting Senior Research Fellow in the School of Healthcare, University of Leeds; KK is a Research Nurse at PMSK; AN is a GP and Director of PMSK Ltd; TP is a Research Assistant in Statistics at the South East Wales Trials Unit.

## Pre-publication history

The pre-publication history for this paper can be accessed here:

http://www.biomedcentral.com/1472-6963/14/160/prepub
